# The effect of maternal anthropometric characteristics and social factors on gestational age and birth weight in Sudanese newborn infants

**DOI:** 10.1186/1471-2458-8-244

**Published:** 2008-07-18

**Authors:** Eltahir M Elshibly, Gerd Schmalisch

**Affiliations:** 1 Departments of Paediatrics and Child Health, University of Khartoum, Sudan; 2 Clinic of Neonatology (Campus Charité Mitte), Universitätsmedizin Berlin, Germany

## Abstract

**Background:**

In Africa low birth weight (LBW) (<2500 g), is the strongest determinant of infant morbidity and mortality. The aim of this study was to quantify the effect of maternal anthropometry, education and socio-economic status on gestational age and birth weight.

**Methods:**

In 1000 Sudanese mothers with singleton births, anthropometric measurements (weight, height, mid-arm circumference) and newborn birth weight were taken within 24 hours of delivery. Furthermore, maternal education and socio-economic status were recorded. The effect of these maternal variables on gestational age and birth weight was investigated by receiver operating characteristic (ROC) curves and by multivariate logistic regression analysis.

**Results:**

Although maternal height was significantly correlated (p = 0.002) with gestational age, we did not find maternal characteristics of value in determining the risk for preterm birth. Birth order was the strongest determinant of birth weight compared to other maternal characteristics. The LBW rate of first born babies of 12.2% was nearly twice that of infants of multiparous mothers. Maternal age and all maternal anthropometric measurements were positively correlated (p < 0.001) with birth weight. A maternal height of <156 cm, a maternal weight of <66 kg, a maternal mid arm circumference of <27 cm and years of education of ≤ 8 years were found to increase the relative risk of LBW but this was statistically significant only in the case of maternal height. Maternal age and BMI had no statistically significant effect on determining the risk for LBW. The social class did not affect the birth weight, while the number of years of education was positively correlated with birth weight (p = 0.01). The LBW rate decreased from 9.2% for ≤ 8 years of education to 6.0% for >12 years of education.

**Conclusion:**

Birth order and maternal height were found to be the most important maternal parameters which influences birth weight and the risk for LBW. The duration of maternal education and not social class was found to significantly affect the risk for LBW.

## Background

There is a large body of literature showing that the world wide problem of low birth weight (LBW), i.e. infants weighing <2500 g, is among the strongest determinants of infant mortality and morbidity. While in industrialized countries the majority of LBW infants do well, thanks to the advances of modern obstetric and neonatal care [1] the chances for intact survival of LBW infants is much lower in African and other developing countries due to inadequate or limited medical care including proper antenatal care [2,3].

Beside biological factors like gestational age (GA), maternal weight and height [4], life style factors such as dietary habits, tobacco, alcohol or caffeine consumption [5] can influence birth weight. Furthermore there are socio-demographic and socio-economic factors that are known to affect birth weight. An example is the work by Wasunna et al. [6] who found that maternal education and household income were important factors affecting birth weight.

In Africa there are much higher percentages of women with low education, poverty and poor nutritional status who are therefore at increased risk of adverse reproductive outcomes including LBW and preterm birth. The identification during pregnancy of such mothers is therefore important in order to determine the level of care and priorities for referral to centres where reasonable obstetric and neonatal care are available. Therefore the aim of this study was to investigate the influence of parity, maternal anthropometry, education and socio-economic status on gestational age and birth weight in a sample of mothers and infants from an inner urban area of Khartoum, Sudan. No such comprehensive study was performed before in Sudan.

## Methods

### Patients

The study was conducted during a one year period and anthropometric measurements were taken from 1000 mothers and their newborns in the Soba University Hospital in Khartoum, Sudan. The mothers were recruited from a large inner urban area of Khartoum with wide differences in the socio-economic status. The three social classes (low, middle, high) were determined by the area of residence. In this study only mothers who were sure of the last date of their menstrual period were included in the study. Mothers who were not sure of their dates or who had multiple pregnancy or had their pregnancy complicated by diabetes mellitus were not included in the study.

The study was approved by the Department of Paediatrics of the University of Khartoum and consent was obtained from the mothers.

### Protocol

In order to exclude inter-observer variation the measurements were taken within 24 hours of birth by one investigator (EME) in the postnatal wards. Maternal anthropometry included mother weight, height and mid arm circumferences. Mothers weight was measured by a standard scale to the nearest 100 grams. The mothers' height was measured with a standard scale for height to the nearest millimeter and the maternal mid arm circumference was measured by an inelastic tape to the nearest millimeter. Babies' weight was measured by a standard scale (Atom Medical, Tokyo, Japan) to the nearest 10 grams, The gestational age was calculated from the last menstrual period in completed weeks of gestation.

### Statistics

Means and standard deviations (SD) were calculated for all maternal anthropometric parameters, gestational age and birth weight. The relationship between maternal anthropometric parameters, gestational age and birth weight was investigated by correlation analysis. Receiver operating characteristic curves (ROC) were drawn to determine optimal cut-off values of the maternal anthropometric parameters that can point to the relative risk for LBW. The optimal cut-off points are defined by the highest numbers of correct classifications considering the LBW rate. The 95% confidence intervals of the area under the normalized ROC curve (AUC) were calculated as described by Hanley and McNeil [7]. Analysis of variance (ANOVA) was used to investigate the effect of education and social class on gestational age and birth weight. A multivariate logistic regression analysis with backward selection to identify significant influencing factors was performed to investigate the effect of maternal characteristics on preterm birth and LBW. Statistical analysis was performed using the software MEDCALC (Version 9.1.0.1, MedCalc Software, Mariakerke Belgium) and Statgraphics Centurion (Version 15, Stat Point Inc., Herndon, Virginia, USA). Statistical significance was defined as a p value <0.05.

## Results

### Characteristics of the mothers and their newborns

Maternal age, anthropometric measurements (body weight, height and mid arm circumference), number of years of education and social status of 1000 Sudanese women and the characteristics of their newborns are shown in Table 1. The number of the first born babies was 370 (37%) and the parity of the 630 (63%) multiparous women ranged from 2 to 14. Most mothers (64.4%) belonged to low social class and a considerable number of women (38.0%) had an education ≤ 8 years. The gestational age (GA) ranged between 28 and 42 complete gestational weeks and the birth weight ranged between 800 and 5100 g. 57 (5.7%) infants were delivered before 37 completed gestational weeks (preterm infants) and 83 (8.3%) infants were of LBW (<2500 g).

**Table 1:** Age, anthropometric parameters, years of education, social class of the mothers and the anthropometric parameters of their newborns (Presented are means (SD), range and N(%))

	Mean (SD) or N(%)	Range
*Mothers*		
Age (years)	27.0 (5.4)	16 to 52
Body weight (kg)	65.2 (13.0)	33.5 to 109.9
Body Height (cm)	159.6 (6.2)	139.5 to 195.5
Mid arm circumference (cm)	26.9 (3.9)	17.0 to 40.9
Body mass index (kg/m^2^)	25.5 (4.8)	13.5 to 47.1
Number of years of education	9.1 (4.3)	0 to 19
Social class		
low	644 (64.4%)	
middle	313 (31.3)	
high	43 (4.3)	
		
*Newborns*		
Gestational age (weeks))	39.1 (1.8)	28 to 42
Birth weight (g)	3131.7 (538.9)	800 to 5100
Boys	514 (51.4%)	
LBW (< 2500 g)	83 (8.3%)	
Preterm infants (<37 weeks)	57 (5.7%)	

### Effect of parity on gestational age and birth weight

**Figure 1:**
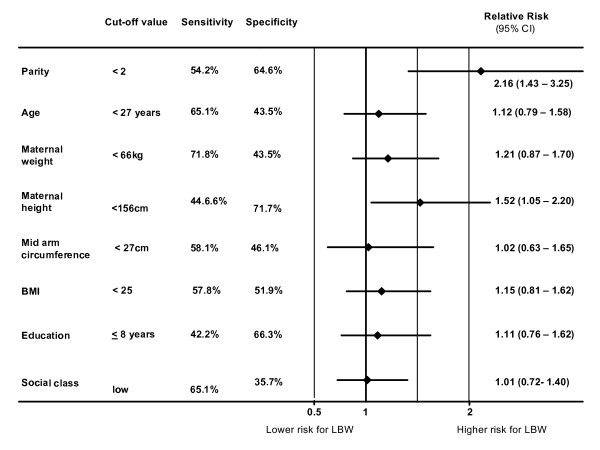
Effect of maternal characteristics on the relative risk for LBW.

With increasing birth order (parity) birth weight increased significantly (p < 0.0001) as shown in Table 2. However increasing birth order did not seem to affect gestational age at all. The LBW rate of first born babies of 12.2% was nearly twice that of infants of multiparous mothers. Fig. 1 illustrates that the birth order is the strongest determinant of the relative risk for LBW compared to all other maternal parameters investigated in this study

**Table 2:** Effect of birth order on gestational age and birth weight

	First birth (N = 370)	Second birth (N = 206)	≥ Third birth (N = 424)	p-value
Gestational age (weeks)	39.1 (1.8)	39.1 (1.7)	39.0 (1.8)	p = 0.573
Birth weight (g)	3021.6 (527.2)	3156.9 (497.1)	3215.7 (553.0)	**p = 0.0001**
LBW-rate (%)	12.2%	5.3%	6.4%	**p = 0.003**

### Influence of maternal anthropometry on gestational age and birth weight

The correlation coefficients between maternal quantitative characteristics (age, body weight, height and mid arm circumference), gestational age and birth weight are shown in Table 3. Obviously, among the maternal characteristics maternal height was the only one that was weakly positively correlated with gestational age (p = 0.002).

**Table 3:** Pearson correlation coefficients (p-values in brackets) between maternal characteristics (age, anthropometry) and gestational age and birth weight (Statistically significant values are printed in bold)

Maternal characteristics	Gestational age	Birth weight
Age	-0.029 (p = 0.355)	**0.108 (p < 0.001)**
Body weight	0.040 (p = 0.211)	**0.165 (p < 0.001)**
Body Height	**0.101 (p = 0.002)**	**0.149 (p < 0.001)**
Mid arm circumference	0.074 (p = 0.098)	**0.171(p < 0.001)**
Body mass index	-0.003(p = 0.938)	**0.112 (p < 0.001)**

In contrast to the gestational age all maternal characteristics were significantly positively correlated (p < 0.001) with birth weight. Therefore, ROC curves were drawn and an optimal cut-off point for each parameter was defined as shown in Table 4. The discriminative power of the maternal characteristics to estimate the risk for LBW was assessed by the area under the curve (AUC). As shown in Table 4 only maternal height and body weight had a statistically significant discriminative ability to distinguish between normal and LBW infants. Sensitivity and specificity of the defined cut-off points are shown in Fig. 1.

**Table 4:** ROC analysis of maternal age and maternal anthropometric parameters in the estimation of the risk for LBW.

Parameter	Optimal cut-off point	AUC with 95%CI	p-value
Maternal age (years)	27	0.536 (0.504 to 0.567)	0.268
Maternal height (cm)	156	0.591 (0.560 to 0.622)	**0.003**
Maternal weight (kg)	66	0.567 (0.536 to 0.599)	**0.037**
Maternal mid arm circumference (cm)	27	0.542 (0.497 to 0.586)	0.351
Body mass index (kg/m^2^)	25	0.535 (0.504 to 0.567)	0.276

The p-value calculated according Hanley and McNeil [7] indicates whether the area under the normalized ROC curve (AUC) is statistically different from 0.5 (no discrimination). If the p value is statistically not significant then there is no evidence that the parameter has the ability to influence the risk for LBW.

Using the cut-off points shown in Table 4 the influence of maternal characteristics on birth weight was investigated by calculation of the relative risk for LBW (Fig. 1). Obviously, if the measurements of maternal characteristics are below the cut off point there is a trend to increase the risk of LBW. However, maternal height was the only anthropometric parameter which was statistically significant. A maternal height <156 cm increases the relative risk for LBW about 52%

### Effect of education and social class on gestational age and birth weight

**Figure 2:**
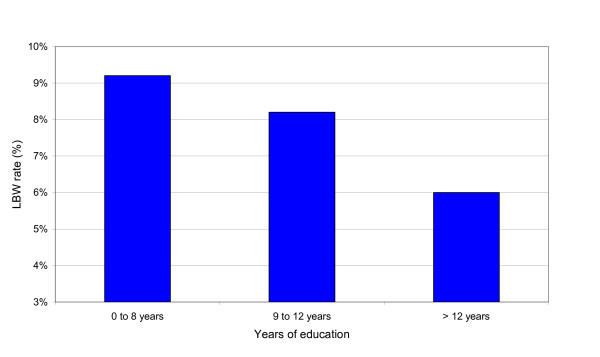
Low birth weight rate in relation to number of years of education.

**Figure 3:**
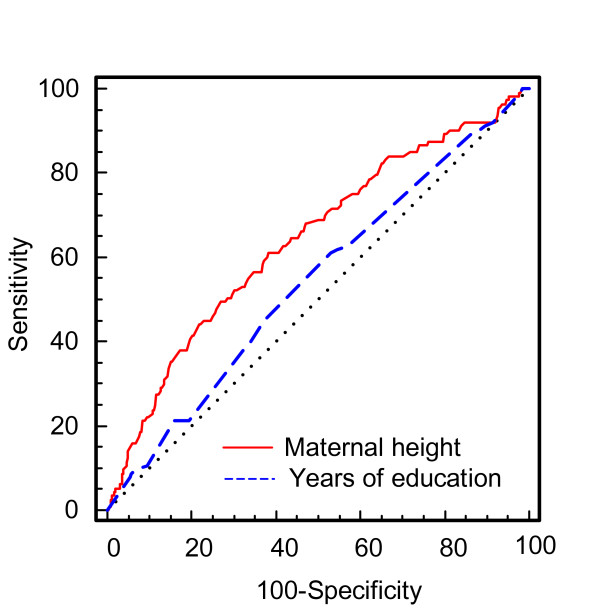
**Comparison of the ROC curves of the maternal height and the years of education to estimate the risk for LBW.** The dotted diagonal line represents "no discrimination". AUC of education is distinctly lower compared to the AUC of maternal height.

The effect of social class and education on gestational age and birth weight is shown in Table 5 and Table 6, respectively. The social class had no statistically significant effect on gestational age and birth weight, while the number of years of education had a statistically significant effect on birth weight but not on gestational age. With increase in the number of years of education the LBW rate decreases (Fig. 2) but the differences were not statistically significant. Furthermore, Fig. 3 shows that the influence of maternal height is distinctly greater than the effect of years of education in determining the risk of LBW.

**Table 5:** Effect of social class on gestational age and birth weight tested by ANOVA

	High social class	Middle social class	Low social class	p-value
	(N = 43)	(N = 313)	(N = 644)	
Gestational age (weeks)	39.1 (1.2)	39.1 (1.9)	39.0 (1.7)	p = 0.777
Birth weight (g)	3208.1 (590.7)	3164.9 (541.9)	3110.5 (533.5)	p = 0.218

**Table 6:** Effect of the number of years of education on gestational age and birth weight (ANOVA, statistically significant p-values are printed in bold)

	0 – 8 years	9 – 12 years	>12 years	p-value
	(N = 380)	(N = 487)	(N = 133)	
Gestational age (weeks)	39.1 (1.9)	39.0 (1.8)	39.2 (1.3)	p = 0.505
Birth weight (g)	3078.2 (552.0)	3139.3 (520.1)	3257.1 (550.8)	**p = 0.004**

### Logistic regression analysis

Additionally to the univariate analysis the effect of birth order, maternal anthropometry and the years of education on preterm birth and LBW was investigated using a multivariate logistic regression model. The evaluation showed that no statistically significant model could be obtained (p = 0.163) describing the influence of maternal characteristics on the probability of preterm birth p (GA<37 weeks).

In contrast to the gestational age a statistically significant model (p = 0.0005) for the probability of LBW (p (BW ≤2500 g)) was found:

$\text{p}(\text{BW}\leq 2500\text{g})=\frac{1}{1+{\text{e}}^{-\text{z}}}$ with

z = 16.90-1.303 · PAR + 0.060 · MA + 0.122 · MW - 0.123 · MH - 0.050 · MMAC - 0.153 · BMI - 0.0340 · YED

Where PAR is the dichotomized parity (=0 for first birth order, =1 for birth order ≥ 2), MA is the maternal age in years, MW is the maternal weight in kg, MH is the maternal height in cm, MMAC is the maternal mid arm circumference in cm and YED is number of years of education. However, some parameters in this model were strongly correlated with each other (e.g., MW and BMI with r = 0.921) so that they did not give additional information and were therefore eliminated by the backward selection method. The backward selection of this model showed that the only statistically significant predictors for LBW were parity (p = 0.009) and maternal height (p = 0.006) and the logistic regression model can be simplified to:

$\text{p}(\text{BW}\leq 2500\text{g})=\frac{1}{1+{\text{e}}^{-\text{z}}}$ with

z = 6.434-0.625 · PAR - 0.054 · MH.

This model shows that the probability of LBW is mainly influenced by birth order and maternal height and this result agrees well with the results of the univariate evaluation shown in Fig. 1.

## Discussion

The status of the mother nutrition and socio-economic variables have long been known to influence the reproductive performance and outcome and the condition of the infant at birth.

In our study using both univariate analysis and confirming by multivariate logistic regression model, we could demonstrate that birth order, maternal anthropometric characteristics and education are of value in estimating the increased risk for LBW. However we could not demonstrate that maternal characteristics can predict the increased risk for preterm delivery as already shown by Voigt et al. [4] et al in Germany and Honest et al. [8] in Great Britain. Beside antenatal care and woman's health status, birth order was found to be one of the major factors affecting birth weight [9], and several studies have shown that birth weight increases with birth order [10-12]. Hirve et al. [13] in India found a 1.3 higher relative risk for LBW in primipara and in Africa Lawoyin [14] found that first born babies had a 3.1 fold higher mortality risk. In our study primiparity was associated with an increased relative risk for LBW of 2.16, and that was distinctly higher when compared to the relative risk for LBW of other maternal characteristics (Fig. 1).

Maternal height was the second most important parameter which influences the risk for LBW in our mothers. Our cut off point of 156 cm agrees well with investigators in Bangladesh [15] who also found that maternal height below 155 cm increases the risk for child death. This confirms the value of maternal height as a predictor of childhood morbidity and mortality. Zhang et al. [16] suggested that the slower fetal growth due to short maternal stature appears to be physiologic. Veena et al. [17] pointed out that the size of the infant at birth is influenced by paternal rather than maternal height while Voigt et al. [4] found that the influence of paternal characteristics on infant size at birth is negligible.

The lower predictive value of maternal weight measured at delivery in our study could be due to the high individual differences in the changes that occur in the body weight during pregnancy. In contrast maternal height is not liable to such changes as a result of pregnancy.

The duration of maternal education and not maternal social class was found to significantly affect the risk for LBW. Karim et al. [18] found that birth weight increases with higher maternal education while in Germany [19] women with the lowest education had significantly elevated risk for small for gestational age newborns (SGA).

Our study confirms the usefulness of anthropometric measurements in identifying mothers at high risk of delivering LBW infants as found by other workers [20,21]. However we would like to point to the limitation of using anthropometric measurements taken before pregnancy to estimate the risk for LBW, as such measurements can seldom be taken in Africa, where women commonly present to health facilities only when they are advanced in pregnancy.

## Conclusion

In our study birth order was found to be the major factor affecting LBW rate. The influence of the other maternal characteristics is distinctly lower. Nevertheless women with a height of <156 cm and education of ≤ 8 years had a LBW rate of 13.7%. This means that almost one seventh of such mothers will have low birth weight babies. Therefore, we recommend that policy makers should make more emphasis on education as it imparts knowledge and thus modify dietary habits and quality of food consumed. This will lead to a better nutritional status in adolescent girls, resulting in lower rates of LBW and hence great reduction in infant morbidity and mortality.

## Abbreviations

AUC: Area under the ROC curve; CI: Confidence interval: GA: Gestational age; LBW: Low birth weight; ROC: Receiver operatic characteristic; SD: Standard deviation.

## Competing interests

The authors declare that they have no competing interests.

## Authors' contributions

EME and GS had primary responsibility for writing of the manuscript. EME carried out all anthropometric measurements and GS performed statistical analysis. All authors read and approved the final manuscript.

## Pre-publication history

The pre-publication history for this paper can be accessed here:


